# Effects of Different Varieties of *Camellia oleifera* on Root-Associated Bacterial Community Structure and Co-Occurrence Network

**DOI:** 10.3390/biology15010071

**Published:** 2025-12-30

**Authors:** Jiechen Zhou, Xiang Duan, Jiao Peng, Tiancai Zhu, Yuanhao He, Guoying Zhou, Junang Liu

**Affiliations:** 1 Hunan Provincial Key Laboratory for Control of Forest Diseases and Pests, Key Laboratory of National Forestry and Grassland Administration for Control of Diseases and Pests of Southern Plantation, Key Laboratory of Cultivation and Protection for Non-Wood Forest Trees, Ministry of Education, Central South University of Forestry and Technology, Changsha 410004, China; christu25@163.com (J.Z.);; 2 Xiangtan Forestry Science Institute, Xiangtan 411206, China

**Keywords:** *Camellia oleifera*, root, bacteria, community structure, co-occurrence network

## Abstract

This study investigates the bacterial community structure and diversity across different root compartments (non-rhizosphere soil, rhizosphere soil, rhizosphere, and endosphere) of *Camellia oleifera* and their associations with three cultivars (‘Huashuo’, ‘Huajin’, ‘Huaxin’). High-throughput sequencing and bioinformatics analyses were performed to characterize the bacterial communities. A total of 22 phyla, 59 classes, 155 orders, 268 families, 523 genera, 929 species, and 2045 operational taxonomic units (OTUs) were identified. Alpha diversity indices (Shannon, Simpson, Chao1) showed no statistically significant differences among the three cultivars, but varied significantly across root compartments. The rhizosphere exhibited the highest bacterial diversity and richness, which was significantly higher than that in the endosphere. At the phylum level, Proteobacteria, Chloroflexi, Actinobacteriota, Acidobacteriota, Firmicutes, and Bacteroidetes dominated the communities. Significant differences were observed in the relative abundance of dominant genera (e.g., Proteus, actinomycetes) among varieties and root compartments. PCoA analysis revealed that ‘Huaxin’ had a distinct bacterial community structure compared to ‘Huashuo’ and ‘Huajin’, while the endosphere was separated from other compartments. Interaction network analysis indicated that most bacterial interactions were positive, with *Colidextribacter*, *Uliginosibacterium*, and *Aliidongia* showing the highest centrality, suggesting their key roles in maintaining community stability. This study provides novel insights into the distribution patterns and driving factors of root-associated bacteria in *C. oleifera*, laying a theoretical foundation for future research on disease control and quality improvement of this crop.

## Simple Summary

*Camellia oleifera* is an important woody oil crop in China. Root-associated bacteria play critical roles in plant growth and health. This study investigated the bacterial community structure and co-occurrence networks in non-rhizosphere soil, rhizosphere soil, rhizosphere, and endosphere of three *C. oleifera* varieties (‘Huashuo’, ‘Huajin’, ‘Huaxin’) using high-throughput sequencing. The results showed that root compartments significantly affected bacterial diversity and composition, with the rhizosphere having the highest diversity. Varietal differences altered the bacterial community structure, especially for ‘Huaxin’, which harbored a distinct community. *Acidothermus*, *Sphingomonas*, and *Actinospica* were identified as key taxa maintaining community stability. This study provides insights into the plant-microbe interactions in *C. oleifera* and a theoretical basis for microbiota-based crop management.

## 1. Introduction

*Camellia oleifera* Abel, a member of the genus Camellia within the Theaceae family, stands as one of the world’s most important woody oil plants, boasting a cultivation history in China that spans over 2300 years [1]. This species thrives in subtropical hilly regions, favoring warm and humid climates [2]. In China, *C. oleifera* is predominantly cultivated south of the Yangtze River, with major production areas in provinces such as Hunan, Jiangxi, and Guangxi [3]. Hunan Province is the leading producer, with a planting area reaching 13.79 million hectares, which constitutes one-third of China’s total planting area [4]. The region’s subtropical monsoon climate, characterized by warm, humid conditions, low mountains, hilly terrain, and slightly acidic soil, provides ideal conditions for extensive cultivation [5].

The health and productivity of plants are deeply intertwined with their associated microorganisms [6]. Root-associated microbes, in particular, form complex symbiotic relationships with plants, utilize root exudates and assist in nutrient conversion, thereby promoting plant growth, adaptation, and biodiversity [7], influencing nutrient acquisition, stress tolerance, and overall fitness [8]. The assemblage of these microbial communities is not random but is influenced by factors such as soil type, climate, and plant genotype [9,10]. For instance, Peiffer et al. [10] demonstrated that the host genotype significantly influences the maize rhizosphere microbiome, while Wang et al. [11] identified notable differences in microbial community structure between rhizosphere and non-rhizosphere soils of *Panax quinquefolius* L. at various growth stages, highlighting a rhizosphere effect. Furthermore, distinct microbial niches exist from the bulk soil to the root endosphere, with a general trend of decreasing diversity from the rhizosphere soil towards the root interior, as observed in rice by Eisen et al. [12].

Despite its economic importance, research on the *C. oleifera* microbiome remains limited, with most studies focusing on fungal communities or the impact of specific diseases. A critical knowledge gap exists regarding how different *C. oleifera* varieties, each with unique genetic and physiological traits, shape their root-associated bacterial communities. Understanding this relationship is essential for harnessing the potential of the microbiome for crop improvement. Based on evidence from other crops, we hypothesize that different *C. oleifera* varieties host distinct root-associated bacterial communities, which are also strongly structured by the root compartment. employs Illumina MiSeq high-throughput sequencing technology to examine the bacterial community structure and diversity in the rhizosphere soil, rhizosphere, and endosphere of various *C. oleifera* varieties. The primary objectives of this study were to: (1) characterize the bacterial community structure and diversity across different root zones of *C. oleifera*; (2) determine the influence of plant variety on these communities; and (3) identify potential keystone taxa within the bacterial co-occurrence networks. Key terms in this study are defined as follows: (1) Non-rhizosphere soil: soil not adhering to roots and without direct interaction with root exudates; (2) Rhizosphere soil: soil adhering to roots within ~1 mm; (3) Rhizosphere: the root surface and the immediate soil interface; (4) Endosphere: the internal tissues of roots.

## 2. Materials and Methods

### 2.1. Overview of the Experimental Site

The experiment was conducted at the Xiangtan Forestry Research Institute Nursery (112°42′ E, 27°51′ N; elevation: 71.13 m), Hunan Province, China. The site experiences a subtropical monsoon humid climate, with an annual average temperature of 16.7–18.3 °C and precipitation of 1300 mm. Soil physicochemical properties (3 replicates per plot) were measured via standard methods: pH (potentiometric method, soil–water = 1:2.5) 5.3 ± 0.2; organic matter (potassium dichromate oxidation) 2.8% ± 0.3; total nitrogen (Kjeldahl method) 0.15% ± 0.02; available phosphorus (Olsen method) 12.5 ± 1.3 mg/kg; available potassium (flame photometry) 85.3 ± 4.2 mg/kg All plants were cultivated under natural field conditions without controlled environmental interventions.

### 2.2. Sample Collection and Processing

In May 2023, non-rhizosphere soil, rhizosphere soil, rhizosphere, and endosphere samples were collected from three *C. oleifera* varieties (‘Huashuo’, ‘Huajin’, ‘Huaxin’) at the experimental site. For each variety, 9 healthy plants with consistent growth were randomly selected. Roots (diameter < 1 mm) were collected at a depth of 5–20 cm and 1 m from the trunk. Non-rhizosphere soil was collected from the same plot, 20 cm away from the plant roots (to avoid root exudate influence). Rhizosphere soil was defined as soil adhering to roots within ~1 mm, collected by gently brushing the root surface.

Sample processing for composite samples: (1) Non-rhizosphere soil: 3 samples from each variety were randomly mixed into 1 composite sample, with 3 replicates (total 3 samples); (2) Rhizosphere soil: 3 samples from 3 plants of the same variety were mixed into 1 composite sample, 3 replicates per variety (total 9 samples); (3) Rhizosphere and endosphere: 3 root samples from 3 plants of the same variety were mixed into 1 composite sample, 3 replicates per variety (total 18 samples). A total of 30 samples were obtained (3 non-rhizosphere + 9 rhizosphere soil + 9 rhizosphere + 9 endosphere). All samples were stored in sterile sealed bags on ice and transported to the laboratory within 4 h.

Root surface sterilization and endosphere isolation: (1) Roots were cleaned with sterile brushes to remove loose soil, then placed in sterile tubes with 10 mL of 0.1 M potassium phosphate buffer (pH 8.0) and vigorously shaken for 5 min (twice). (2) Roots were transferred to 50 mL vials with 20 mL phosphate buffer and ultrasonicated for 10 min (40 kHz, 100 W). The combined wash solutions were filtered through a 0.22 μm membrane to collect rhizosphere bacteria [13]. (3) Surface sterilization: Washed roots were soaked in 70% ethanol for 30 s, followed by 5% sodium hypochlorite for 5 min, then rinsed 5 times with sterile water. The final rinse water was plated on LB medium to check for sterility (no colonies indicated successful sterilization) [14]. (4) Sterilized roots were stored at −80 °C for endosphere bacterial analysis, while rhizosphere membranes and soil samples were stored at −80 °C.

### 2.3. DNA Extraction and PCR Amplification

DNA was extracted from 0.5 g soil samples and 0.2 g root samples using the Power Soil^®^ DNA Isolation Kit (Qiagen, Hilden, Germany). DNA purity (A260/A280 ratio: 1.8–2.0) and concentration (≥50 ng μL^−1^) were determined by agarose gel electrophoresis (1%) and a NanoDrop 2000 spectrophotometer (Thermo Fisher Scientific, Waltham, MA, USA). The V5–V7 variable region of the 16S rRNA gene was selected for amplification due to its high resolution for rhizosphere bacterial communities. Primer pairs 799F (5′-AACMGGATTAGATACCCKG-3′) and 1193R (5′-ACGTCATCCCCACCTTCC-3′) were used. PCR reactions were performed in 25 μL volumes containing 1 μL DNA template (50 ng μL^−1^), 12.5 μL 2×Taq PCR MasterMix, 1 μL each primer (10 μM), and 9.5 μL sterile water. Positive controls (*E. coli* DH5α DNA) and negative controls (sterile water) were included. PCR conditions: initial denaturation at 95 °C for 3 min; 35 cycles of 94 °C for 50 s, 58 °C for 60 s, 72 °C for 60 s; final extension at 72 °C for 10 min. PCR products were verified by 1% agarose gel electrophoresis. Purification was performed using the Qiagen PCR Purification Kit (Qiagen, Hilden, Germany), and concentrations were quantified with a Qubit 3.0 fluorometer (Thermo Fisher Scientific, Waltham, MA, USA). Purified products were pooled in equimolar amounts and sequenced on the Illumina MiSeq PE250 platform (Illumina, San Diego, CA, USA) with dual-index barcoding. Demultiplexing was performed using Illumina bcl2fastq software (v2.20) [15].

### 2.4. High-Throughput Sequencing Data Analysis

Raw paired-end reads were processed using fastp software (v0.19.6) for quality control: trimming reads with tail-end quality scores < 20, applying a 50 bp sliding window (average quality < 20 was trimmed), removing reads < 50 bp and those containing N bases. FLASH software (v1.2.11) was used for read merging with a minimum overlap length of 10 bp and a maximum mismatch ratio of 0.2 in the overlap region [16].

UPARSE software (v7.1) was used to cluster sequences into OTUs at a 97% similarity threshold, and chimeras were removed using UCHIME. Chloroplast and mitochondrial sequences were excluded. Sequences from each sample were rarefied to 20,000 reads (based on the minimum sequencing depth across samples) to standardize sequencing effort. The average sequence coverage per sample was 99.09% [17].

Taxonomic annotations were assigned using the RDP classifier (v2.11) against the Silva 16S rRNA gene database (v138, 2021) with a confidence level of 70% [18]. Community composition was summarized at the phylum, class, order, family, and genus levels [19].

### 2.5. Data Statistics

Statistical analyses were performed using SPSS v19.0 and R software (v3.6.3) [20].

(1) Alpha diversity analysis

Alpha diversity indices (Shannon, Simpson, Chao1) were calculated using mothur software (v1.44.3). Differences in alpha diversity among groups were tested using one-way ANOVA followed by Tukey’s HSD test (*p* < 0.05).

(2) Beta diversity analysis

Beta diversity was analyzed using principal coordinate analysis (PCoA) based on Bray–Curtis distances. PERMANOVA (permutational multivariate analysis of variance) was performed to test differences in community structure among groups (999 permutations, *p* < 0.05).

(3) Identification of dominant taxa

Dominant taxa were defined as those with a relative abundance > 5% (phylum level) or >1% (genus level). Linear discriminant analysis effect size (LEfSe) was used to identify differentially abundant taxa (LDA score > 3.0, *p* < 0.05).

(4) Co-occurrence network analysis

Spearman’s correlation analysis was performed to construct co-occurrence networks (|r| > 0.6, *p* < 0.05). Network topology parameters (nodes, edges, density, modularity, clustering coefficient) were calculated using Networkx software (v2.6.3) [21]. Centrality indices (degree, closeness, betweenness) were used to identify key taxa. Network stability was evaluated by sequentially removing the 50 least abundant nodes and comparing changes in centrality indices.

## 3. Results

### 3.1. Sequencing Data Analysis

**Figure 1:**
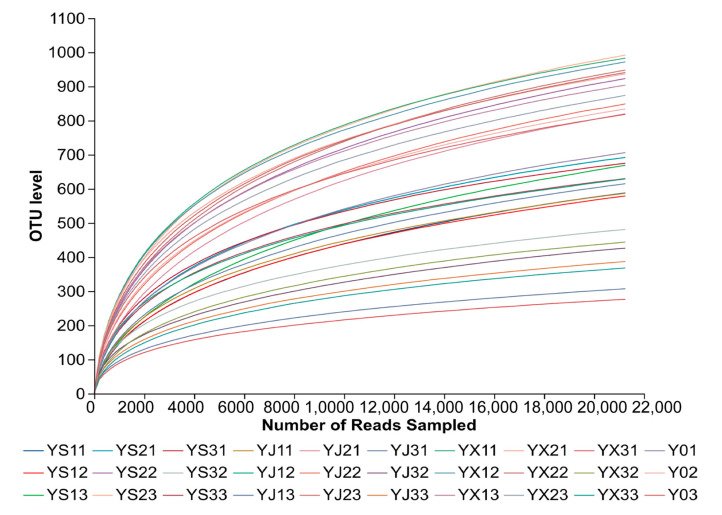
Rarefaction curves of all samples.

High-throughput sequencing of 30 samples (3 non-rhizosphere soil, 9 rhizosphere soil, 9 rhizosphere, 9 endosphere) generated 1,511,857 valid reads, with an average of 50,395 reads per sample. The rarefaction curves approached saturation, indicating sufficient sequencing depth (Figure 1). Sequencing was performed on the Illumina MiSeq PE250 platform, with a read length of 250 bp. OTUs were clustered at 97% similarity using UPARSE, and 2045 OTUs were obtained after removing chimeras, chloroplast, and mitochondrial sequences. After rarefaction to 20,000 reads, the average sequence coverage was 99.09%, and rare OTUs (<0.01% relative abundance) were removed.

### 3.2. Analysis of Alpha Diversity of Bacterial Communities in the Root System of C. oleifera

We compared the alpha diversity of bacterial communities in the root systems of different *C. oleifera* varieties, as shown in Table 1. The Shannon, Simpson, and Chao1 indices revealed differences in the richness and diversity of bacterial communities among the three varieties.

Alpha diversity indices of root-associated bacterial communities among different *C. oleifera* varieties and root compartments are shown in Table 1. Among the three varieties, no statistically significant differences were observed in Shannon, Simpson, or Chao1 indices (*p* > 0.05). For root compartments, the Shannon index was significantly higher in the rhizosphere (4.721 ± 0.4369) than in the endosphere (3.76 ± 0.4649) (*p* < 0.05). The Simpson index was significantly lower in the rhizosphere (0.03574 ± 0.02696) than in the endosphere (0.07092 ± 0.02582) (*p* < 0.05), indicating higher community evenness in the rhizosphere (since lower Simpson index values reflect higher evenness). The Chao1 index was significantly higher in the rhizosphere (1202 ± 75.14) than in the endosphere (630.5 ± 154.3) (*p* < 0.05), suggesting higher species richness in the rhizosphere.

The Shannon, Simpson, and Chao1 indices showed no significant differences among the bacterial communities in the root systems of various *C. oleifera* varieties. However, significant differences emerged in the Shannon and Simpson indices when comparing the rhizosphere and endosphere bacterial communities across different root compartments. The Chao1 index revealed significant differences between the endosphere and the other three compartments, as well as between the rhizosphere soil and the rhizosphere bacterial community.

### 3.3. Analysis of the Basic Composition and Structure of Bacterial Communities in the Root System of C. oleifera

**Figure 2:**
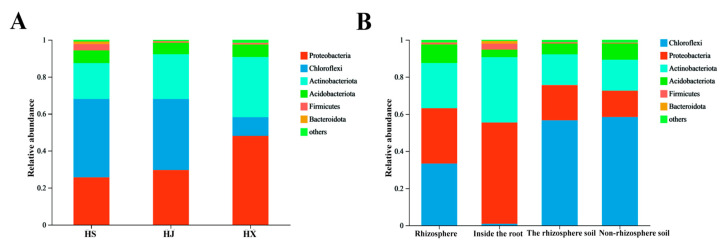
Relative abundance of the root-associated bacterial communities in different *Camellia oleifera* varieties at the phylum level. (**A**) Comparison among different varieties; (**B**) Comparison among different root compartmen.

**Figure 3:**
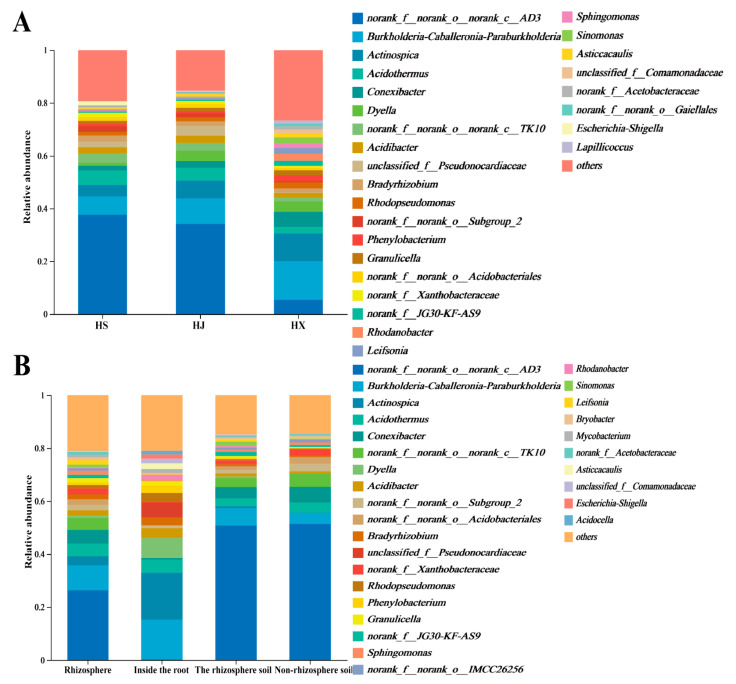
Relative abundance of the root-associated bacterial communities in different *Camellia oleifera* varieties at the genus level. (**A**) Comparison among different varieties; (**B**) Comparison among different root compartmen.

We analyzed the composition and relative abundance of bacterial communities in the root-associated microbiota of *C. oleifera* at the phylum and genus taxonomic levels (Figure 2 and Figure 3). Bacterial groups with a relative abundance of less than 1% in the samples were categorized as “other”.

At the phylum level, the bacterial communities within the root system primarily consist of six phyla: Proteobacteria, Chloroflexi, Actinobacteria, Acidobacteriota, Firmicutes, and Bacteroidetes.

The root bacterial communities of various *C. oleifera* varieties share dominant bacterial groups, including Proteobacteria, Chloroflexi, Actinobacteria, and Acidobacteriota. Despite this commonality, the relative abundances of these phyla differ among the three varieties. In ‘Huaxin’ *C. oleifera*, Proteobacteria exhibited the highest relative abundance at 48.0%. In contrast, Chloroflexi reached up to 40% in both ‘Huashuo’ and ‘Huajin’ *C. oleifera*, while it constituted only 10% in ‘Huaxin’ (Figure 2A).

The composition and relative abundance of dominant bacterial groups in *C. oleifera* vary across different root compartments. In non-rhizosphere soil, rhizosphere soil, and the rhizosphere, the dominant bacterial groups include Chloroflexi, Proteobacteria, Actinobacteria, and Acidobacteriota. In contrast, the endosphere is primarily dominated by Proteobacteria and Actinobacteria. Notably, Chloroflexi constitutes less than 1% of the endosphere bacterial community, a lower proportion compared to the other two root compartments. Firmicutes are prevalent in the endosphere, accounting for 54.5% of the bacterial community, whereas they represent only 14.2% in rhizosphere soil. The relative abundance of Chloroflexi decreases progressively from rhizosphere soil to the rhizosphere and endosphere. In contrast, the relative abundance of Proteobacteria and Actinobacteria exhibits a significant decreasing trend from the interior to the exterior (Figure 2B).

At the genus level, excluding Norank and Unclassified groups, differences exist in the composition and relative abundance of dominant bacterial groups within the root bacterial communities across different samples.

Among the various *C. oleifera* varieties, ‘Huashuo’ and ‘Huajin’ share dominant bacterial groups, including *Burkholderia–Caballeronia–Paraburkholderia*, *Actinospica*, and *Acidothermus*. In contrast, ‘Huaxin’ *C. oleifera* comprises *Burkholderia–Caballeronia–Paraburkholderia*, *Actinospica*, and *Conexibacter*. The relative abundances of these genera differ among the three varieties. Notably, *Burkholderia–Caballeronia–Paraburkholderia* and *Actinospica* exceed 10% in relative abundance in ‘Huaxin’, which is higher than in ‘Huashuo’ and ‘Huajin’ (Figure 3A).

In various root compartments, distinct bacterial communities are observed. The endosphere is primarily populated by *Burkholderia–Caballeronia–Paraburkholderia*, *Actinospica*, *Acidothermus*, and *Dyella*. In contrast, the rhizosphere is dominated by *Burkholderia–Caballeronia–Paraburkholderia* and *Conexibacter*, while the rhizosphere soil is predominantly occupied by *Burkholderia–Caballeronia–Paraburkholderia* alone. Notably, *Conexibacter* constitutes less than 1% of the endosphere bacterial community, which is lower than its presence in the rhizosphere bacterial community. *Actinospica* represents 17.59% of the endosphere samples but is found at less than 1% in the rhizosphere soil. *Dyella* is a leading genus in the endosphere samples, yet it accounts for less than 1% in the other two root compartments. Additionally, the relative abundance of *Burkholderia–Caballeronia–Paraburkholderia* increases progressively from the rhizosphere soil to the rhizosphere and then to the endosphere (Figure 3B). *Burkholderia–Caballeronia–Paraburkholderia* is a monophyletic group with similar ecological functions, so it was analyzed as a composite genus.

### 3.4. Differences in Bacterial Community Structure of C. oleifera Root Systems

**Figure 4:**
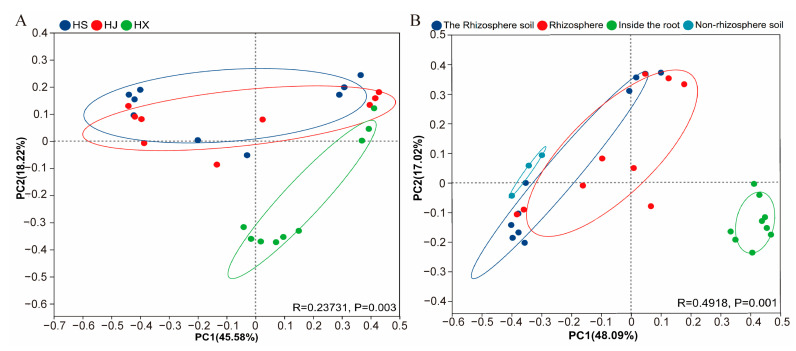
PCoA and PERMANOVA analysis represent community compositional dissimilarity of root-associated bacteria among different varieties. (**A**) Comparison among different varieties; (**B**) Comparison among different root compartmen.

PCoA analysis based on Bray–Curtis distances showed that the bacterial community of ‘Huaxin’ was significantly separated from ‘Huashuo’ and ‘Huajin’ (PERMANOVA, R = 0.2373, *p* = 0.003), while no significant difference was observed between ‘Huashuo’ and ‘Huajin’. This indicates that the variety significantly influenced the bacterial community structure of *C. oleifera* root systems, with an explanatory rate of 45.58% (Figure 4A).

For root compartments, the endosphere community was significantly separated from other compartments (PERMANOVA, R = 0.4918, *p* = 0.001), while no significant difference was found between rhizosphere and rhizosphere soil. Furthermore, a separation was noted between the non-rhizosphere soil and root bacterial communities. These findings indicate that different root zones significantly influenced the bacterial community structure of *C. oleifera* root systems, accounting for 48.09% of the variation (Figure 4B).

**Figure 5:**
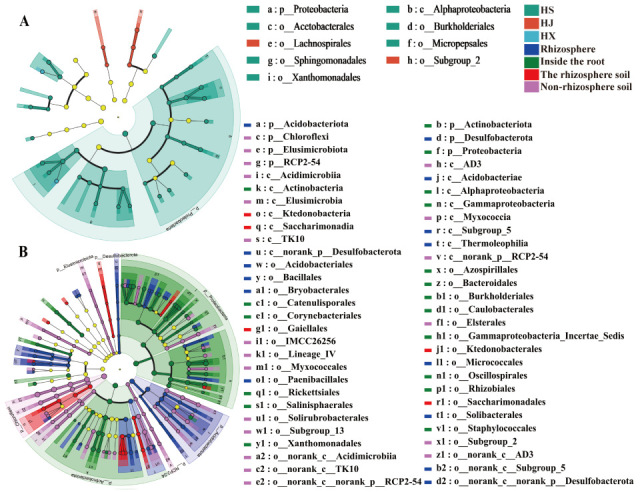
LEfSe analysis of root-associated bacterial community structures in different varieties. (**A**) Comparison among different varieties; (**B**) Comparison among different root compartmen.

We employed LEfSe analysis to identify taxa with significantly different abundances and used Linear Discriminant Analysis (LDA) to assess the impact of each species’ relative abundance on these differences. Our findings revealed that the diameter of each circle is proportional to the relative abundance of the corresponding taxon. The circles, arranged from inner to outer, represent taxonomic levels from phylum to genus. Only taxa with LDA scores exceeding 3 are shown (Figure 5A,B).

At the detectable genus level, excluding Norank and Unclassified taxa, bacterial taxa with LDA scores greater than 3 varied across samples. Specifically, ‘Huashuo’ had 1 distinct genus, ‘Huajin’ had 2, and ‘Huaxin’ had 12. In the rhizosphere, there were 11 distinct genera, the endosphere had 19, rhizosphere soil had 4, and non-rhizosphere soil had 6. Among the bacterial communities of the three *C. oleifera* varieties, ‘Huashuo’ and ‘Huajin’ exhibited the fewest different genera, whereas ‘Huaxin’ had the most. The number of distinct genera in the bacterial communities decreased progressively from the inner to the outer regions of the root system.

**Figure 6:**
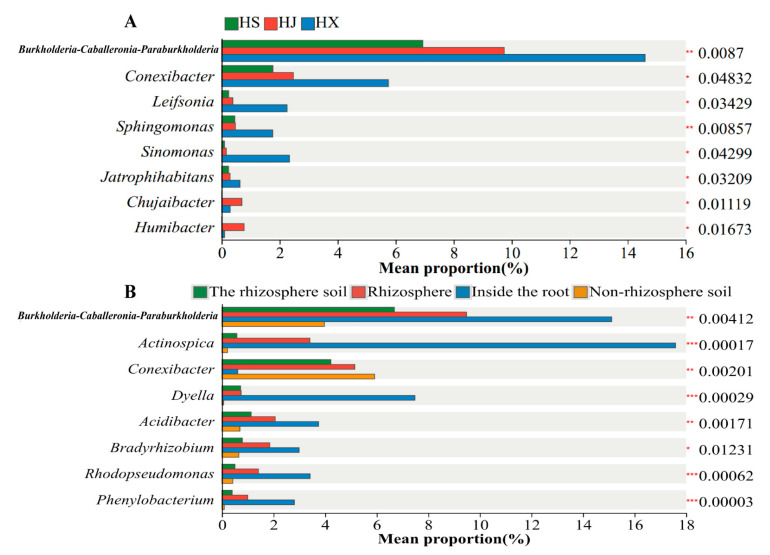
Significance test of intergroup differences among samples at the genus level. The numbers on the far right are the *p*-values, with * *p* < 0.05, ** *p* < 0.01, *** *p* < 0.001. (**A**) Comparison among different varieties; (**B**) Comparison among different root compartmen.

The intergroup significance test results indicated that, at the genus level (excluding Norank and Unclassified taxa), the top eight most abundant bacterial taxa in the root bacterial communities of different *C. oleifera* varieties were *Burkholderia–Caballeronia–Paraburkholderia*, *Conexibacter*, *Leifsonia*, *Sphingomonas*, *Sinomonas*, *Jatrophihabitans*, *Chujaibacter*, and *Humibacter*. The relative abundances of these taxa varied across samples. Notably, *Burkholderia–Caballeronia–Paraburkholderia* and *Sphingomonas* exhibited highly significant differences (*p* < 0.01), whereas the other six genera showed significant differences (*p* < 0.05) (Figure 6A).

In examining the bacterial communities across various root zones of *C. oleifera*, the top eight most abundant genera, excluding Norank and Unclassified taxa, were identified. These genera and their relative abundances in each sample included *Burkholderia–Caballeronia–Paraburkholderia*, *Actinospica*, *Conexibacter*, *Dyella*, *Acidibacter*, *Rhodopseudomonas*, *Phenylobacterium*, and *Granulicella*, which demonstrated extremely significant differences (*p* < 0.01) (Figure 6B).

### 3.5. C. oleifera Root Bacterial Interaction Network

**Figure 7:**
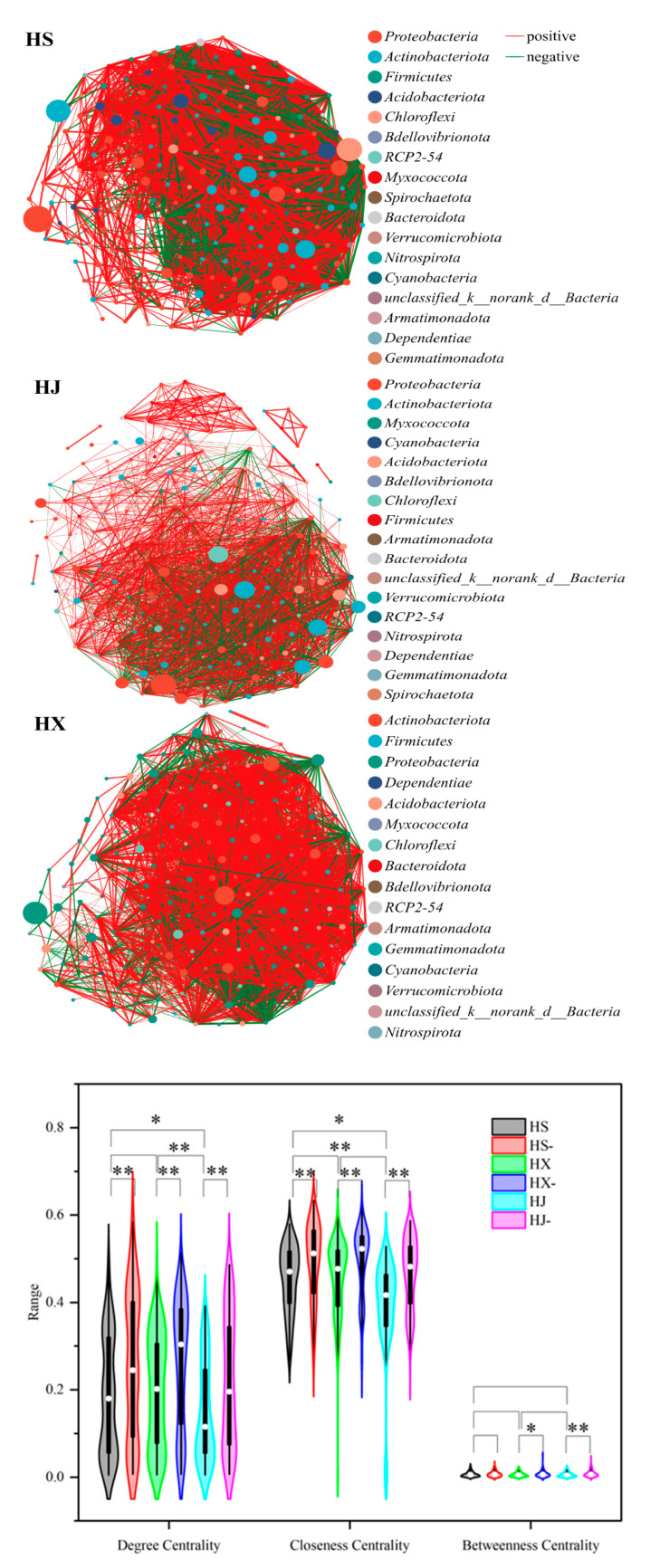
Analysis of root-associated bacterial interaction network at the genus level in different varieties. Significance: * *p* < 0.05; ** *p* < 0.01.

Co-occurrence networks were constructed based on Spearman’s correlation analysis of genus-level data (|r| > 0.6, *p* < 0.05) (Figure 7). The network topology analysis revealed distinct characteristics for each *Camellia oleifera* variety. ‘Huaxin’ network exhibited the highest density at 0.197265, followed by ‘Huashuo’ network at 0.182663, while ‘Huajin’ network was the sparsest at 0.148291; their modularity values were extremely close to 0 (‘Huaxin’: 0.003395, ‘Huajin’: 0.035889, ‘Huashuo’: 0.008702), far below the threshold of 0.3 required for significant community clustering. The rhizosphere bacterial interaction network of ‘Huashuo’ consisted of 198 nodes and 3634 connections, with 2123 positive and 1511 negative correlations. In contrast, the ‘Huaxin’ network comprised 194 nodes and 3693 connections, featuring 2800 positive and 893 negative correlations. Meanwhile, ‘Huajin’ included 200 nodes and 2951 connections, with 1968 positive and 983 negative correlations. Among the varieties, ‘Huaxin’ exhibited higher connectivity due to its fewer nodes but more edges. Within these networks, *Colidextribacter*, *Uliginosibacterium*, and *Aliidongia* were the most connected species in ‘Huashuo’, ‘Huaxin’, and ‘Huajin’, respectively.

The species exhibiting high centrality and correlation varied across the three networks. In the ‘Huashuo’ network, the bacterial genera with the highest centrality coefficients were *Colidextribacter*, *Aliidongia*, *Bifidobacterium*, *Romboutsia*, and *Acidicapsa*. In contrast, the ‘Huaxin’ network featured *Uliginosibacterium*, *Ralstonia*, *Acidothermus*, *Occallatibacter*, and *Acidicapsa* as the top genera. Meanwhile, in the ‘Huajin’ network, the leading genera were *Asticcacaulis*, *Aliidongia*, *Bacillus*, *Nitrospira*, and *Paenibacillus*. These species likely play a crucial role in maintaining the stability of the microbial ecological network.

Network stability was evaluated by removing the 50 least abundant nodes (consistent with the average number of rare genera across networks). Initially, no significant differences in centralities were found between ‘Huashuo’ and ‘Huaxin’, whereas ‘Huajin’ exhibited notable differences in degree and closeness centrality compared to the other two varieties. Post-removal, all three varieties showed significant changes in degree and closeness centrality. In terms of betweenness centrality, ‘Huashuo’ experienced minimal change, ‘Huaxin’ showed significant differences, and ‘Huajin’ displayed highly significant differences. These results suggest that the rhizosphere bacterial network of ‘Huashuo’ is more stable, while ‘Huajin’ is the least stable.

## 4. Discussion

Roots host a diverse microbial ecosystem, where rhizosphere microorganisms and their environment form a complex system essential for plant growth and development. Research indicates that the structure and diversity of plant root microbial communities are shaped by factors such as plant species, host genotype, season, geographical location, and environmental conditions. *C. oleifera*, a significant oilseed plant, has primarily been studied concerning rhizosphere fungi and the impact of external interventions on microbial communities. However, research on its root bacterial communities remains limited [22,23,24]. To gain deeper insights into the root bacterial communities of *C. oleifera*, this study examined the diversity and community structure of rhizosphere soil, rhizosphere, and endorhizosphere bacterial communities across three different *C. oleifera* varieties. The findings revealed that while bacterial community diversity and richness were generally consistent across different *C. oleifera* varieties, there were notable differences in diversity and richness among various root zones.

Compared to other rhizosphere compartments, the bacterial community structure and composition within plant roots are highly specific, with a notable reduction in bacterial diversity. This suggests that the plant root surface selectively influences which bacteria enter the rhizosphere [25,26]. Lundberg [26] examined the rhizosphere microbial communities of 600 Arabidopsis thaliana samples and discovered that the Shannon diversity in the rhizosphere was significantly lower than in the rhizosphere soil community. Similarly, Yuan et al. [27] found that microbial diversity within the roots of Paeonia lactiflora was considerably lower than in the rhizosphere and non-rhizosphere environments. In our study, the bacterial community diversity and richness within the roots of *C. oleifera* were significantly lower than those in the rhizosphere and rhizosphere soil, indicating that only a subset of bacteria can colonize and thrive in the rhizosphere environment of *C. oleifera*. Furthermore, most microorganisms enriched in the endorhizosphere are also present in the rhizosphere and non-rhizosphere environments [28]. At the phylum level, Proteobacteria and Actinobacteria dominate the bacterial communities in *C. oleifera* across different root zones. Additionally, bacteria with low abundance in the rhizosphere tend to exhibit low abundance in the endorhizosphere as well. In summary, the root surface acts as a filter, screening bacteria for colonization within the root. The number and diversity of bacteria in the rhizosphere and rhizosphere soil exceed those in non-rhizosphere environments, influenced by root exudates from plant root metabolism. For instance, in poplar plantations, rhizosphere bacterial diversity is higher than in non-rhizosphere environments [29]. Peiffer et al. [10] also observed significant differences in the diversity and richness of microbial communities between the rhizosphere and surrounding soil of maize, with higher microbial activity in the rhizosphere. However, in this study, while rhizosphere bacterial diversity was higher than in rhizosphere soil and non-rhizosphere soil, rhizosphere soil bacterial diversity was lower than in non-rhizosphere soil.

Plant variety significantly influences the structure of root bacterial communities. ‘Huashuo’, ‘Huajin’, and ‘Huaxin’ exhibited low similarity in their bacterial communities, suggesting that different *C. oleifera* varieties impact their rhizosphere bacterial communities differently, even under identical environmental conditions. This phenomenon is not unique to *C. oleifera* and aligns with previous findings in other plants. For instance, Li et al. [30] identified differences in the rhizosphere bacterial community structure among four pepper cultivars, while Liu et al. [31] reported variations in soil fungal diversity and community composition among pecan cultivars. ‘Huaxin’ harboring a distinct community. This may be attributed to differences in root exudate composition among varieties [32].

At the phylum level, Proteobacteria and Actinobacteriota dominated all compartments, reflecting their adaptability to root environments. Proteobacteria possess an outer membrane primarily composed of lipopolysaccharides, which safeguard their genetic material from external disturbances [33]. Actinobacteriota produce antibiotics and promote plant growth, contributing to disease resistance [34]. Dominant bacterial groups are crucial in managing ecological functions [32]. This group includes various pathogenic and nitrogen-fixing bacteria, demonstrating strong environmental adaptability and the ability to rapidly colonize the rhizosphere of diverse plants [35]. For instance, *Burkholderia* can colonize plant surfaces and rhizospheres, fix nitrogen, solubilize phosphorus, reduce plant ethylene levels, and produce auxins [36]. Actinomycetes and their derivatives not only promote plant growth but also serve as biopesticides. More than half of the over 10,000 antibiotics produced by microorganisms originate from Actinomycetes [37]. Previous research on plant leaf and root microbial communities has identified Proteobacteria, Actinobacteria, Bacteroidetes, and Firmicutes as stable components of plant microbiomes [38]. Besides the two dominant groups, Chloroflexi exhibited notable variations in bacterial communities across different root zones. Chloroflexi plays a crucial role in plant CO_2_ fixation and has been shown to interact closely with other microorganisms within the community [39,40,41]. This is mainly because, apart from the root interior, other root zones are exposed to the external environment, allowing Chloroflexi to become a dominant group in these root communities.

Co-occurrence network analysis revealed dominant positive interactions, indicating cooperative relationships (e.g., nutrient exchange) among bacteria [42]. All three networks fall into the “sparse network” category (density < 0.2), which aligns with the typical structural pattern of natural microbial ecosystems where species interactions are selective rather than random. ‘Huaxin’ network had the highest connectivity (99.0% of nodes in one component) and average degree (38.07), reinforcing its role as the most interconnected system. ‘Huajin’ network, despite its lowest density, maintained a high connectivity rate (94.5%) and an average degree of 29.51, suggesting efficient information transfer despite fewer total interactions. ‘Huashuo’ network stood out for its balanced positive-negative interaction ratio: with 58.4% positive edges (synergistic relationships) and 41.6% negative edges (competitive/inhibitory relationships), it had a higher proportion of negative interactions than ‘Huaxin’ and ‘Huajin’, indicating more intense interspecific competition in the ‘Huashuo’ environment. These findings indicate that the root bacterial community of *C. oleifera* is rich in probiotics that contribute to metabolizing various nutrients in the roots, supplying essential nutrients and hormones for plant growth and development. Key taxa with high centrality (e.g., *Acidothermus*, *Sphingomonas*) are involved in organic matter decomposition and stress resistance [43,44]. *Acidothermus* degrades cellulose, providing carbon sources for other bacteria [43], while *Sphingomonas* enhances plant drought resistance [44]. These taxa are potential candidates for developing microbial inoculants to improve *C. oleifera* productivity.

This study has limitations: (1) It was conducted at a single site and time, so future studies should include multiple sites and seasons; (2) Root exudate composition was not analyzed, which would help explain varietal differences; (3) Functional verification of key taxa was not performed. Future research should focus on isolating key bacteria and testing their effects on *C. oleifera* growth and disease resistance. Additionally, breeding programs could consider selecting varieties with beneficial microbial associations to enhance sustainable production.

## 5. Conclusions

This study characterized the root-associated bacterial communities in three *C. oleifera* varieties across four root compartments. The main findings are as follows: (1) Root compartments significantly affected bacterial diversity and composition, with the rhizosphere having the highest diversity and the endosphere the lowest. The rhizosphere presumably acts as a gateway for bacteria entering plant interiors, exerting a filtering effect; (2) Varietal differences altered bacterial community structure, with ‘Huaxin’ having a distinct community; (3) Proteobacteria, Chloroflexi, and Actinobacteriota were the dominant phyla, while *Colidextribacter*, *Uliginosibacterium*, and *Aliidongia* were key genera; and (4) Co-occurrence networks were dominated by positive interactions, with *Acidothermus*, *Sphingomonas*, and *Actinospica* maintaining community stability. The identified key taxa and network characteristics offer a basis for developing microbial inoculants to improve *C. oleifera* growth and disease resistance.

## Figures and Tables

**Table 1:** Alpha diversity of root-associated bacterial communities in different *Camellia oleifera* varieties. Different letters following the numbers indicate statistically significant differences (*p* < 0.05, ANOVA).

Category	Name	Shannon Index	Simpson Index	Chao1 Index
Varieties	HS	4.132 ± 0.572 a	0.06721 ± 0.04066 a	970.8 ± 208.2 a
	HJ	4.037 ± 0.5267 a	0.05599 ± 0.02771 a	881 ± 261.2 a
	HX	4.465 ± 0.8597 a	0.04368 ± 0.03546 a	991.8 ± 344.1 a
Root Compartments	Rhizosphere	4.721 ± 0.4369 bA	0.03574 ± 0.02696 bA	1202 ± 75.14 B
	Endosphere	3.76 ± 0.4649 a	0.07092 ± 0.02582 a	630.5 ± 154.3 a
	Rhizosphere Soil	4.153 ± 0.7249 ab	0.06022 ± 0.04318 ab	1011 ± 166.4 A
	Non-rhizosphere Soil	4.378 ± 0.3395 ab	0.05196 ± 0.02056 ab	1093 ± 99.14 AB

## Data Availability

The original contributions presented in this study are included in the article. Further inquiries can be directed to the corresponding authors.
